# Recruitment and Baseline Characteristics of Participants in the Finnish Geriatric Intervention Study to Prevent Cognitive Impairment and Disability (FINGER)—A Randomized Controlled Lifestyle Trial †

**DOI:** 10.3390/ijerph110909345

**Published:** 2014-09-10

**Authors:** Tiia Ngandu, Jenni Lehtisalo, Esko Levälahti, Tiina Laatikainen, Jaana Lindström, Markku Peltonen, Alina Solomon, Satu Ahtiluoto, Riitta Antikainen, Tuomo Hänninen, Antti Jula, Francesca Mangialasche, Teemu Paajanen, Satu Pajala, Rainer Rauramaa, Timo Strandberg, Jaakko Tuomilehto, Hilkka Soininen, Miia Kivipelto

**Affiliations:** 1 Department of Chronic Disease Prevention, National Institute for Health and Welfare, P.O. Box 30, FI-00271 Helsinki, Finland; E-Mails: jenni.lehtisalo@thl.fi (J.Le.); esko.levalahti@thl.fi (E.L.); tiina.laatikainen@thl.fi (T.L.); jaana.lindstrom@thl.fi (J.Li.); markku.peltonen@thl.fi (M.P.); satu.ahtiluoto@fimnet.fi (S.A.); antti.jula@thl.fi (A.J.); miia.kivipelto@ki.se (M.K.); 2 Alzheimer’s Disease Research Center, Karolinska Institutet, Novum 5th floor, SE-14157 Stockholm, Sweden; 3 Institute of Public Health and Clinical Nutrition, University of Eastern Finland, P.O. Box 1627, FI-70211 Kuopio, Finland; 4 Department of Neurology, Institute of Clinical Medicine, University of Eastern Finland, P.O. Box 1627, FI-70211 Kuopio, Finland; E-Mails: alina.solomon@uef.fi (A.S.); hilkka.soininen@uef.fi (H.S.); 5 Aging Research Center, Karolinska Institutet—Stockholm University, Gävlegatan 16, SE-11330 Stockholm, Sweden; E-Mail: Francesca.Mangialasche@ki.se; 6 Institute of Health Sciences/Geriatrics, University of Oulu, and Oulu University Hospital, P.O. Box 5000, FI-90014 University of Oulu, Finland; E-Mails: riitta.antikainen@ouka.fi (R.A.); timo.strandberg@oulu.fi (T.S.); 7 Medical Research Center Oulu, Oulu University Hospital and University of Oulu, P.O. Box 5000, FI-90014 University of Oulu, Finland; 8 Oulu City Hospital, Kiviharjuntie 5, FI-90015 Oulu, Finland; 9 Department of Neurology, Kuopio University Hospital, P.O. Box 100, FI-70029 KYS, Kuopio, Finland; E-Mail: tuomo.hanninen@kuh.fi; 10 Finnish Institute of Occupational Health, Topeliuksenkatu 41 a A, FI-00250 Helsinki, Finland; E-Mail: teemu.paajanen@ttl.fi; 11 Department of Lifestyle and Participation, National Institute for Health and Welfare, P.O. Box 30, FI-00271 Helsinki, Finland; E-Mail: satu.pajala@thl.fi; 12 Kuopio Research Institute of Exercise Medicine, Kuopion yliopisto, Haapaniementie 16, 70100 Kuopio, Finland; E-Mail: rainer.rauramaa@uef.fi; 13 Department of Medicine, Geriatric Clinic, University of Helsinki, and Helsinki University Central Hospital, P.O. Box 22, FI-00014 University of Helsinki, Finland; 14 Department of Public Health, University of Helsinki, P.O. Box 40, FI-00014 University of Helsinki, Finland; E-Mail: jaakko.tuomilehto@thl.fi; 15 South Ostrobothnia Central Hospital, Huhtalantie 53, FI-60220 Seinäjoki, Finland; 16 Center for Vascular Prevention, Danube-University Krems, Dr.-Karl-Dorrek-Str. 30, 3500 Krems, Austria; 17 Diabetes Research Group, King Abdulaziz University, P.O. Box 80200, Jeddah 21589, Saudi Arabia

**Keywords:** cognitive impairment, dementia, Alzheimer’s disease, lifestyle, intervention, randomized controlled trial

## Abstract

Our aim is to describe the study recruitment and baseline characteristics of the Finnish Geriatric Intervention Study to Prevent Cognitive Impairment and Disability (FINGER) study population. Potential study participants (age 60–77 years, the dementia risk score ≥6) were identified from previous population-based survey cohorts and invited to the screening visit. To be eligible, cognitive performance measured at the screening visit had to be at the mean level or slightly lower than expected for age. Of those invited (n = 5496), 48% (n = 2654) attended the screening visit, and finally 1260 eligible participants were randomized to the intervention and control groups (1:1). The screening visit non-attendees were slightly older, less educated, and had more vascular risk factors and diseases present. The mean (SD) age of the randomized participants was 69.4 (4.7) years, Mini-Mental State Examination 26.7 (2.0) points, systolic blood pressure 140.1 (16.2) mmHg, total serum cholesterol 5.2 (1.0) mmol/L for, and fasting glucose 6.1 (0.9) mmol/L for, with no difference between intervention and control groups. Several modifiable risk factors were present at baseline indicating an opportunity for the intervention. The FINGER study will provide important information on the effect of lifestyle intervention to prevent cognitive impairment among at risk persons.

## 1. Introduction

With the aging population, it has been projected that the number of persons with cognitive impairment and dementia will increase rapidly in the coming years [1]. Longitudinal population-based studies have identified many potentially modifiable vascular, metabolic, and life-style related risk factors for dementia and Alzheimer’s disease (AD) [2].

Interventions on modifiable risk factors may prevent/postpone dementia onset, but the pharmacological and non-pharmacological intervention studies conducted so far have had somewhat disappointing results as pointed out in a report by the National Institutes of Health [3]. There are several reasons for this. Previous trials have mostly used a single agent intervention and they have been conducted in older and/or already cognitively impaired populations, which may partly explain the modest results. Many of these trials were planned for other outcomes and cognitive outcomes were secondary. There are however some positive signs that antihypertensive drug treatment [4], vitamin B supplementation [5], physical activity [6] and cognitive training [7] may be beneficial, at least in certain population groups. Given the multifactorial nature of dementia/AD, and the long prodromal period, it has been proposed that the optimal feasible clinical trial should be a multidomain intervention targeting an at-risk population [3]. Such multidomain lifestyle interventions have demonstrated dramatic benefits in the development of type 2 diabetes in high-risk individuals [8].

To interpret the results of the intervention studies and to use the results in developing intervention programs, it is important to understand who participates in these trials, and to what extent the results may be generalizable. Little is known about the determinants of participation in clinical trials among older people, as studies are often conducted at memory clinic settings or among volunteers recruited through advertisements, *etc*. In one cognitive training trial, participants were younger, more likely to be female, and had higher education than the general population of the same age-range [9]. Often these demographic data are the only data available for comparison. In some previous observational studies it has been reported that the non-participants had lower cognitive test performance [10] and higher prevalence of dementia [11]. There are few health promotion and physical activity trials among older adults, however not focusing on AD or cognition, with varying reports on non-participation: the participants have reported more health problems [12] than the non-participants in one study, but better health in another [13]. One study reported that while those non-participants who refused to participate were healthier, those who could not be reached were less healthy than the participants [14].

We have initiated the Finnish Geriatric Intervention Study to Prevent Cognitive Impairment and Disability (FINGER) [15] to investigate whether a multidomain intervention could prevent cognitive decline and eventually dementia. It is a 2-year randomized controlled trial (RCT) targeting several known risk factors simultaneously through an intervention consisting of nutritional guidance, exercise training, cognitive training, and intensive monitoring of vascular risk factors. The participants were recruited from earlier population-based cardiovascular disease and type 2 diabetes (non-intervention) surveys, which gives us unique background information on both participants and those that did not participate. This is rarely available in clinical trials. The aim of this report is two-fold: first we will describe the demographic, medical and lifestyle characteristics of screening visit attendees and non-attendees. Second, we will describe the recruitment process and baseline characteristics and cognitive assessments of the study participants in the intervention and control groups.

## 2. Experimental Section

The protocol of the FINGER study (ClinicalTrials.gov identifier: NCT01041989) has been described earlier [15]. In brief, the FINGER study is an ongoing multi-centre randomized controlled trial. The 2-year multidomain intervention consists of nutritional guidance; exercise; cognitive training and social activity; and management of metabolic and vascular risk factors. Persons in the control group receive regular health advice. The primary outcome is cognitive performance measured with the modified Neuropsychological Test Battery (mNTB). Main secondary outcomes are: dementia (after extended follow-up), disability, vascular risk factors and outcomes, depressive symptoms, quality of life, and neuroimaging measures. The intensive intervention was completed in 2014, and the participants are followed-up for additional five years. The FINGER study has been approved by the Coordinating Ethics Committee of the Helsinki and Uusimaa Hospital District. The participants gave written informed consent at the time of both the screening and baseline visits.

### 2.1. Recruitment

The participants were recruited from persons who had earlier participated in population-based non-communicable disease risk factor surveys: the National FINRISK study [16] in 1972, 1977, 1982, 1987, 1992, 1997, 2002 or 2007, or the Finnish type 2 diabetes prevention program’s population survey [17] in 2004 or 2007. These independent cross-sectional surveys were conducted for health surveillance purposes to monitor chronic disease risk factor levels in the population. The participation rates of these surveys were good, ranging from 70% to above 96% in the birth cohort that was the target of the FINGER study [16,18]. The recruitment of participants was started from the most recent survey, and moved on to earlier surveys when all eligible persons of the most recent survey had been invited.

**Figure 1:**
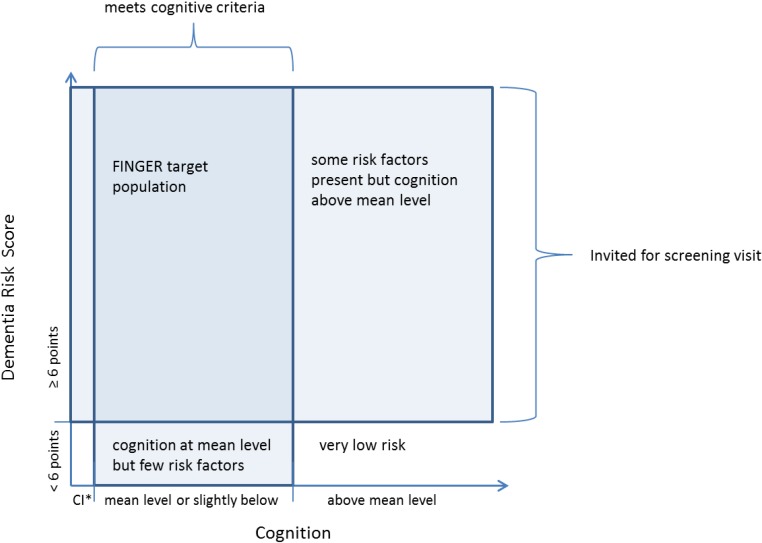
FINGER inclusion criteria and target population.

To be invited, the person had to be aged 60–77 years (born between 1 January 1932 and 31 December 1949) at the beginning of the study, and have Cardiovascular Risk Factors, Aging and Incidence of Dementia (CAIDE) Dementia Risk Score [19] of 6 points or higher, and be alive when the sample was drawn in May 2009. The score includes easily measurable variables (age, sex, education, hypertension, hypercholesterolemia, obesity and physical inactivity) that are associated with the risk of dementia. The 6 points cut-off is relatively low but indicates a presence of some modifiable risk factors. The majority (84%) of our source population met this criterion, excluding only those with very low risk of dementia. At the screening visit participants’ cognition was assessed with The Consortium to Establish a Registry for Alzheimer’s Disease (CERAD) neuropsychological battery [20]. To be included in the FINGER, the participants had to fulfil at least one of the following criteria: (1) Word List Learning task (10 words × 3) ≤19 words; or (2) Word List Recall ≤75%; or (3) Mini Mental State Examination (MMSE) ≤26/30 points. This criteria selects persons with cognitive performance at the mean level or slightly lower than expected for age according to Finnish population norms (inclusion cut-off z-scores −0.5, −0.2 and −0.9 respectively) [21]. Theoretical formation of this at-risk population is presented in Figure 1. Exclusion criteria were conditions affecting engagement in the intervention (especially the exercise component): present malignant diseases, major depression, dementia/substantial cognitive decline according to clinical interview, MMSE < 20, symptomatic cardiovascular disease, re-vascularisation within one year, severe loss of vision, hearing or communicative ability, conditions preventing co-operation as judged by the local study physician, as well as coincident participation in any other intervention trial.

Screening began in September 2009 in the five original study areas in Finland (in and around the cities of Helsinki, Kuopio, Oulu, Seinäjoki, Vantaa), and a sixth area (Turku) was added in April 2010 to ensure sufficient recruitment in a reasonable time frame. Screening was completed in December 2011 when the intended recruitment target (n = 1200) was achieved.

After completing the baseline measurements randomization was performed in blocks of four persons (two persons randomly allocated to each group) at each site by the study nurse running a computer program. If spouses participated in the trial, the randomization status of the latter recruited spouse was manually changed to match the randomization of the first recruited spouse.

### 2.2. Measurements

The baseline study visit comprised of a detailed medical history and physical examination by trained study physicians, and measurements of height (without shoes), weight (in light indoor clothing), waist (midway between the lowest rib and iliac crest) and hip (at the point yielding the maximum circumference over the buttocks) circumference in a standing position, and systolic and diastolic blood pressure (two measurements using a validated automatic device (Microlife WatchBP Office) in a sitting position, using the right arm, after 10 min of rest) by trained study nurses. Mean value of two blood pressure measurements was used. A fasting venous blood sample was taken from all participants, and a 2 h oral glucose tolerance test with a 75 g glucose load was done in the participants without history of diabetes. Fluoride citrate tubes were used for glucose samples. The separated serum and plasma samples were frozen immediately and mailed monthly to the laboratory of the National Institute for Health and Welfare. Total serum cholesterol and plasma glucose concentrations were determined enzymatically using commercial reagents from Abbott Laboratories on a clinical chemistry analyzer, Architect c8000 (Abbott Laboratories, Abbott Park, IL, USA). The participants filled structured questionnaires including questions of sociodemographic factors, health status, lifestyles, mood and quality of life. The Short Physical Performance Battery [22] was administered by trained physiotherapists.

A thorough cognitive assessment using a set of standard neuropsychological tests (an extended and adapted version of the NTB [23]) was administered by trained study psychologists. The primary outcome measure is the total composite mNTB score including 14 tests that form three different cognitive domains. The memory domain included Visual Paired Associates immediate (score range, 018) and delayed (score range, 0–6); Logical Memory immediate (score range, 0–25) and delayed (score range, 0–25) of the Wechsler Memory Scale-Revised (WMS-R) [24]; and Word List Learning (score range, 0–30) and Delayed Recall (score range, 0–10) of the CERAD test battery [20]. The executive function domain included Category Fluency Test [20], Digit Span [24], Concept Shifting Test [25] (condition C), Trail Making Test [26] (shifting score B-A), and a shortened 40-stimulus version of the original Stroop test [27] (interference score 3–2). The processing speed domain included Letter Digit Substitution Test [28], Concept Shifting Test (condition A), and Stroop test (condition 2). All baseline information was collected before randomization.

In addition to the measurements carried out during the screening and baseline visits, we have background information on vascular risk factors and lifestyles available from the earlier background surveys. Data on health status were also collected through computerized register linkage to three nationwide health registers: the Hospital Discharge Register, the National Social Insurance Institution’s Drug Reimbursement Registry, and the Causes of Death registry using the national personal identification number. Data on all patients discharged from all hospitals in Finland have been recorded in a computerized Hospital Discharge Register since 1968. The diagnoses for hospitalizations have been coded according to the International Classification of Diseases (ICD) version 8 during 1968 to 1986, version 9 during 1987 to 1995, and version 10 since 1996. We used data on hospitalisations before the onset of study for myocardial infarction (ICD 8 and 9 diagnoses 410 and ICD-10 diagnoses I21–I22), stroke (ICD 8 diagnoses 430, 431 (except 43101, 43191) 433, 434, 436, ICD 9 diagnoses 430, 431, 4330A, 4331A, 4339A, 4340A, 4341A, 4349A, 436 and ICD 10 diagnoses I60–I64 (not I636)), cancer (ICD 8 and 9 diagnoses 140–172, 174–208 and ICD 10 diagnoses C00–C43, C45–C97), diabetes (ICD 8 and 9 diagnoses 250 and ICD 10 diagnoses E10–E14), and dementia (ICD 8 diagnosis 290 ICD 9 diagnoses 290, 3310, 4378A and ICD 10 diagnoses F00, F01, F02, F03, G30). In the register the diagnoses are assigned by the physician treating the patient. The Social Insurance Institution’s register (reimbursement of pharmaceutical expenses) was used for diabetes (purchases of drugs in Anatomical Therapeutic Chemical (ATC) category A10 or with the Institution’s special reimbursement code for diabetes) and dementia (purchases of drugs in ATC category N06D or with special reimbursement code for dementia). Currently the data from all of these registers is available until end of 2011.

### 2.3. Statistical Analyses

The differences between screening visit attendees and non-attendees, between randomized and not-randomized participants, and between intervention and control groups were analysed using chi square-test and *t*-test as appropriate. The level of significance was 5% in all analyses.

## 3. Results and Discussion

### 3.1. Characteristics of the Persons invited to the FINGER Study

A total of 5496 persons were invited and 2654 (48% of those invited) attended the screening visit. The screening attendance rates between study sites varied from 43% to 57%. Compared with the attendees, those who did not attend the screening visit were older, and based on the data from the earlier studies they had participated in, they were more likely to be less educated, and have more vascular and lifestyle risk factors (Table 1). The screening visit non-attendees also had more vascular diseases, diabetes, cancer and dementia as identified through the registries. Mortality between 22 May 2009 when the study population was identified and the 31 December 2011 (data currently available through registers) was higher among those who did not attend the screening visit. Therefore, it is likely that many non-responding persons would have been excluded based on our exclusion criteria, had they attended the screening visit.

**Table 1:** Background characteristics from earlier surveys and health registries of the persons who were invited to attend the FINGER study screening visit (mean (SD) or %).

Characteristic		All Invited n = 5496	Screened n = 2654	Screening Visit Non-Attendees n = 2842	*p*-Value *
Age at the beginning of the study (07.09.2009)		68.1 (5.0)	67.8 (4.8)	68.3 (5.2)	0.002
Sex (% men/women)		50.3/49.7	48.8/51.2	51.7/48.3	0.037
Study site:					
	Helsinki (n, %)	743	389 (52.3%)	354 (47.6%)	<0.001
	Kuopio (n, %)	1444	625 (43.3%)	819 (56.7%)	
	Oulu (n, %)	420	240 (57.1%)	180 (42.9%)	
	Seinäjoki (n, %)	587	326 (55.5%)	261 (44.5%)	
	Turku (n, %)	1400	656 (46.9%)	744 (53.1%)	
	Vantaa (n, %)	902	418 (46.3%)	484 (53.7%)	
**Demographic and vascular risk factors from the earlier non-intervention survey**					
Time between earlier survey and invitation (years)		14.3 (10.0)	12.6 (9.7)	15.9 (10.1)	<0.001
Education (years)		10.4 (3.6)	10.7 (3.8)	10.0 (3.5)	<0.001
Systolic blood pressure (mmHg)		143 (18.9)	142 (18.7)	144 (19.1)	<0.001
Total cholesterol (mmol/L)		5.9 (1.1)	5.8 (1.1)	6.0 (1.1)	<0.001
Body mass index (kg/m^2^)		27.7 (4.6)	27.7 (4.5)	27.7 (4.7)	0.575
Physical activity at least 2 times/ week (%)		49.9%	52.4	47.5	<0.001
Dementia risk score (points)		8.4 (1.8)	8.1 (1.7)	8.6 (1.8)	<0.001
**History of diseases from registers**					
Myocardial infarction (%)		4.1	3.5	4.6	0.032
Stroke (%)		4.5	3.0	5.8	<0.001
Diabetes (%)		11.0	8.9	12.9	<0.001
Cancer (%)		8.7	7.0	10.3	<0.001
Dementia (%)		1.1	0.3	2.0	<0.001
Mortality between May 2009 and 31 Dec 2011 (%)		3.2	0.8	5.4	<0.001

* The *p* value refers to difference between screening visit attendees and non-attendees.

**Figure 2:**
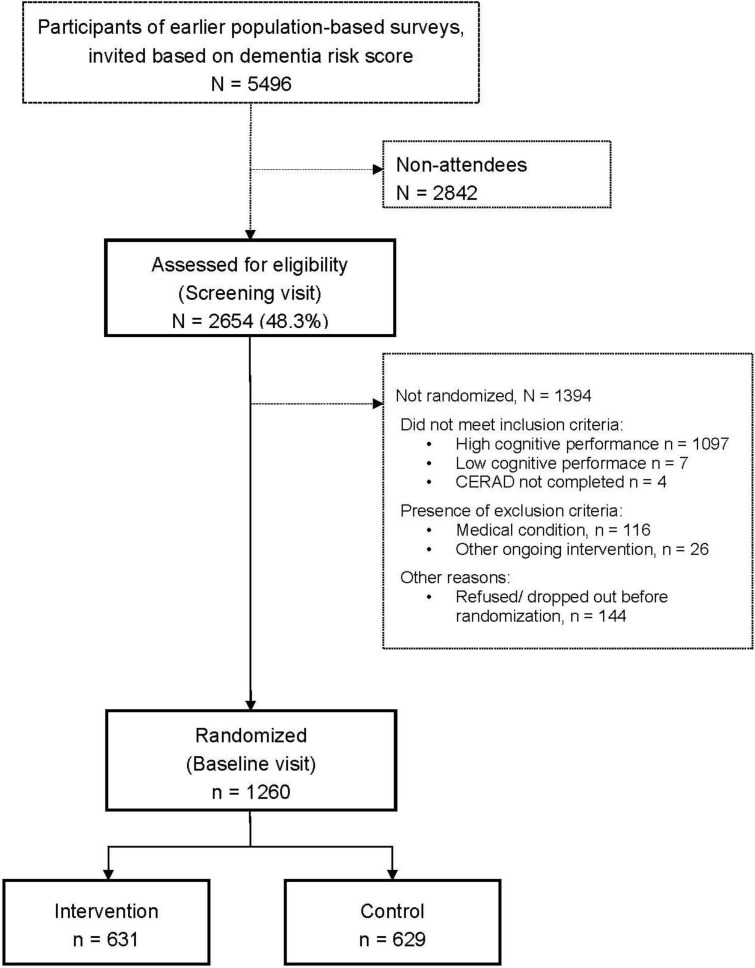
Formation of the study population.

Nearly half of the persons who attended the screening visit fulfilled the cognition criteria for inclusion (Figure 2). The majority of persons who were not included based on their cognitive performance had too high scores, and only seven persons were not included because of too low MMSE score. A total of 142 persons were excluded due to other reasons (too ill including persons suspected to have significant cognitive impairment, participation in another ongoing intervention trial). Additionally 144 persons dropped out after the screening visit but before randomization. This left us with 1260 participants randomized into an intensive multidomain intervention group (intervention, n = 631) and a regular health advice group (control, n = 629).

### 3.2. Randomized vs. not Randomized Persons

The persons who attended the screening visit but who were not randomized were younger, and had higher education, and were less often physically active (based on the data from the background surveys) compared with those who were randomized (Table 2). Blood pressure and serum cholesterol levels were similar in both groups at the time of earlier surveys. Slightly more women than men attended the screening visit but more men than women were eventually randomized. At most study sites, about half of the screened persons were randomized, however, in Turku 36% and Oulu 42% of the screened persons were randomized. As expected, based on the inclusion criteria, cognitive test scores were higher among those that did not fulfil the inclusion criteria.

**Table 2:** Characteristics of the persons who attended the screening visit according to eligibility to the trial (mean (SD) or %).

Characteristic		Randomized	Not Randomized	*p*-Value
		n = 1260	n = 1394	
Age at the screening visit (years)		69.2 (4.7)	68.3 (4.9)	<0.001
Sex (% men/women)		53.3/46.7%	44.8/55.2%	<0.001
Study site:				
	Helsinki (n = 389)	53.5	46.5	<0.001
	Kuopio (n = 625)	55.2	44.8	
	Oulu (n = 240)	41.7	58.3	
	Seinäjoki (n = 326)	49.7	50.3	
	Turku (n = 656)	36.4	63.6	
	Vantaa (n = 418)	49.3	50.7	
**Demographic and vascular risk factors from the earlier non-intervention survey**				
Time between earlier survey and invitation (years)		13.1 (10.1)	12.0 (9.2)	0.003
Education (years)		10.0 (3.3)	11.4 (4.0)	<0.001
Systolic blood pressure (mmHg)		141.8 (18.8)	142.2 (18.5)	0.599
Total cholesterol (mmol/L)		5.8 (1.1)	5.8 (1.1)	0.196
Body mass index (kg/m^2^)		27.6 (4.4)	27.9 (4.6)	0.062
Physical activity at least 2 times/ week (%)		54.6	50.4	0.032
Dementia Risk Score (points)		8.3 (1.8)	8.0 (1.6)	<0.001
**Vascular risk factors and cognitive performance at the FINGER screening visit**				
Systolic blood pressure (mmHg)		143.4 (18.2)	142.8 (17.7)	0.413
Diastolic blood pressure (mmHg)		81.8 (10.1)	81.9 (10.2)	0.808
Body mass index (kg/m^2^)		28.4 (6.2)	28.8 (9.4)	0.189
Mini Mental State Examination (points)		26.7 (2.0)	28.0 (1.9)	<0.001
CERAD Word List Learning (words)		18.4 (3.2)	21.9 (3.5)	<0.001
CERAD Word Recall (words)		5.5 (1.7)	7.5 (1.8)	<0.001
CERAD Category Fluency (words)		21.6 (5.7)	23.7 (6.4)	<0.001

CERAD: Consortium to Establish a Registry for Alzheimer’s Disease.

A majority of not randomized persons were not eligible due to high cognitive performance at the screening visit (n = 1097). Those who were not randomized due to other reasons (n = 297) were older and had lower cognitive performance at the screening visit than the randomized participants, but their vascular characteristics in earlier surveys and at the screening visit did not differ (results not shown).

### 3.3. Randomization into Intervention vs. Control Group

There were no significant differences in sociodemographic, vascular, or lifestyle characteristics between the persons randomized into the intervention and control groups (Table 3). Overall, several vascular risk factors and unhealthy lifestyle factors were present. Nearly 66% of the participants reported history of hypertension and 67% hypercholesterolemia, and 50% had elevated systolic blood pressure and 54% had elevated serum total cholesterol values at the baseline visit. Approximately 30% of the participants were obese, and 71% were engaged in physical activity at least 2 times per week.

**Table 3:** Baseline characteristics of the persons who were randomized to the trial (mean (SD) or %).

Characteristics at Baseline	n	All	Intervention	Control	*p*-Value
		n = 1260	n = 631	n = 629	
**Demographic characteristics**					
Age at the baseline visit (years)	1260	69.4 (4.7)	69.5 (4.7)	69.2 (4.7)	0.27
Sex (men/women, %)	1260	53.3/46.7	54.7/45.3	52.0/48.0	0.34
Education (years)	1244	10.0 (3.4)	10.0 (3.5)	10.0 (3.4)	0.91
Married/ cohabiting (%)	1259	74.3	73.0	75.5	0.31
**Vascular factors**					
Systolic blood pressure (mmHg)	1249	140.1 (16.2)	140.2 (16.7)	140.0 (15.7)	0.75
Systolic blood pressure > 140 mmHg	1249	50.3	50.6	50.0	0.84
Diastolic blood pressure (mmHg)	1249	80.3 (9.5)	80.5 (9.6)	80.1 (9.3)	0.48
Diastolic blood pressure > 90 mmHg	1249	15.0	15.9	14.0	0.33
Serum total cholesterol (mmol/L)	1255	5.2 (1.0)	5.2 (1.0)	5.2 (1.0)	0.93
Serum total cholesterol > 5.0 mmol/L	1255	53.7	53.8	53.6	0.94
Fasting plasma glucose (mmol/L)	1257	6.1 (0.9)	6.1 (0.8)	6.1 (1.0)	0.99
Fasting plasma glucose >7.0 mmol/L	1257	10.5	10.5	10.5	0.98
2 h oral glucose tolerance test (mmol/L)	1085	7.0 (2.2)	7.0 (2.2)	7.0 (2.2)	0.95
2 h oral glucose tolerance test > 11.0 mmol/L	1085	5.6	5.7	5.5	0.90
Body mass index (kg/m^2^)	1249	28.2 (4.7)	28.3 (4.5)	28.1 (4.9)	0.46
Body mass index > 30 kg/m^2^	1249	29.9	29.8	29.9	0.98
Waist circumference (cm)	1250	98.3 (12.6)	98.7 (12.2)	97.9 (13.1)	0.28
**Lifestyles**					
Physical activity at least 2 times/ week (%)	1238	71.0	70.0	72.0	0.46
Current smokers (%)	1214	9.4	10.5	8.3	0.17
Alcohol drinking at least once/week (%)	1250	51.4	51.5	51.2	0.91
Fish intake at least twice/week (%)	1250	52.3	53.2	51.4	0.54
Daily intake of vegetables (%)	1252	61.9	61.5	62.3	0.77
**Self-reported medical conditions**					
Hypertension (%)	1246	65.9	66.7	65.1	0.54
Hypercholesterolemia (%)	1250	67.2	65.4	69.0	0.17
Diabetes (%)	1250	13.1	13.7	12.5	0.52
History of myocardial infarction (%)	1254	5.1	5.2	5.0	0.82
History of stroke (%)	1251	5.4	5.3	5.6	0.79
**Other**					
Dementia risk score	1260	8.3 (1.8)	8.3 (1.8)	8.3 (1.8)	0.92
Short Physical Performance Battery score	1178	10.8 (1.5)	10.8 (1.5)	10.8 (1.5)	0.96

The mean MMSE score was 26.7 (SD 2.0), and it was similar in the intervention and control groups. There were no statistically significant differences on the individual sub-items of the main outcome variable mNTB between the intervention and control groups at baseline (Table 4). On average 77% (SD 22) of the items were remembered on the CERAD word recall test (delayed recall/immediate recall of 3rd list).

**Table 4:** Cognitive performance of the randomized participants and formation of the cognitive outcome modified Neuropsychological Test Battery (mean (SD)).

Characteristics at Baseline		All	Intervention	Control	*p*-Value
	n	n = 1260	n = 631	n = 629	
**Memory**					
WMS-R Logical Memory (immediate)	1258	11.0 (3.7)	10.9 (3.8)	11.1 (3.6)	0.37
WMS-R Logical Memory (delayed)	1257	9.3 (3.9)	9.2 (4.0)	9.5 (3.8)	0.17
CERAD Word List Learning	1257	18.4 (3.2)	18.3 (3.2)	18.6 (3.3)	0.08
CERAD Word List Recall	1255	5.5 (1.7)	5.5 (1.7)	5.6 (1.7)	0.46
WMS-R Visual Paired Associates (immediate)	1239	9.1 (3.8)	8.9 (3.8)	9.3 (3.8)	0.08
WMS-R Visual Paired Associates (delayed)	1237	3.4 (1.8)	3.3 (1.8)	3.4 (1.8)	0.38
**Executive function**					
CERAD Category Fluency	1257	21.6 (5.7)	21.3 (5.6)	21.8 (5.8)	0.13
WMS-R Digit Span (total)	1258	11.5 (2.9)	11.5 (2.9)	11.4 (2.9)	0.64
CST (condition C) *	1157	65.2 (40.6)	64.3 (37.2)	66.1 (43.7)	0.44
TMT shifting score (B-A) *	1180	107.7 (65.7)	110.8 (66.9)	104.6 (64.4)	0.10
Stroop test interference score (3-2) *	1240	34.6 (18.0)	34.8 (18.1)	34.4 (17.8)	0.69
**Processing speed**					
Letter Digit Substitution Test	1253	22.0 (6.0)	21.7 (5.9)	22.2 (6.0)	0.14
CST (condition A) *	1256	33.1 (9.5)	33.3 (8.9)	32.8 (10.0)	0.33
Stroop test (condition 2) *	1251	29.5 (6.4)	29.6 (6.3)	29.3 (6.5)	0.55
Mini Mental State Examination	1257	26.7 (2.0)	26.7 (2.0)	26.8 (2.0)	0.55

* Timed task where smaller number indicates faster performance/better test result. In other tasks bigger number indicates better result. WMS-R: Wechsler Memory Scale-Revised; CERAD: Consortium to Establish a Registry for Alzheimer’s Disease; CST: Concept Shifting Test; TMT: Trail Making Test.

### 3.4. Discussion

The FINGER study is one of the first large multidomain intervention studies aiming at preventing cognitive impairment and dementia. Participants were recruited from earlier non-intervention population-based surveys in Finland, which gives unique background information about the target population typically not available in clinical trials. Analysing these data indicates that persons who did not attend the screening visit of the FINGER study were older, more often men, less educated and had more vascular and lifestyle related risk factors compared to those who were screened. However, baseline clinical characteristics of the participants indicate that several vascular risk factors and unhealthy lifestyle related factors were present, creating a window of opportunity for prevention. The intervention and control groups were similar at the beginning of the trial indicating that the randomization worked well.

Our results are in line with previous epidemiological studies on dementia/AD showing that participants are in general younger, more educated, and healthier than non-participants [9,10,11]. However, very little has been known about participation in prevention trials targeting lifestyle related risk factors among elderly persons. Because the FINGER study includes a long-term and relatively intensive multidomain intervention, it was expected that older persons with worse health status were less likely to participate. To balance the healthy volunteer bias that is often present especially in studies using advertisement, we used the CAIDE Dementia Risk score as one inclusion criteria. We used the cut-off of 6 points or more in the CAIDE risk score to select persons with some modifiable risk factors [15,19]. A large proportion (more than 80%) of the target-aged population met these criteria based on the data from the earlier population-based surveys, and only those with a very low risk of dementia were excluded.

The FINGER population was defined in relation to the goals of the trial and was not intended to be representative of the general population. Nevertheless, the levels of several risk factors in our randomized participants were quite similar to those observed in the general population of the same age: for example in the National FINRISK study in 2012 [29], mean serum total cholesterol level in the Finnish population aged 65–74 years was 5.6 in women and 5.0 mmol/L in men, mean body mass index 28.0 and 28.3 kg/m^2^, and mean systolic blood pressure 146 and 144 mmHg, respectively. However, the proportion of persons with self-reported hypertension and hypercholesterolemia was higher and the proportion of those with regular physical activity was lower in our study population than in the general population of the same age. This is in line with the fact that the CAIDE Dementia Risk Score including the previously mentioned factors was used in recruiting the participants of the FINGER study.

Our inclusion criteria for cognition at the screening visit were used in order to select a high-risk population, and to exclude both the individuals whose cognitive performance was above the mean level for their age and those with substantial cognitive impairment or dementia. While the main reason for non-eligibility was too good cognitive performance, the group of non-randomized persons was still heterogeneous and included also persons with too low cognitive performance, severe diseases or otherwise unable/willing to participate to the trial. As the CAIDE risk score emphasizes vascular risk factors and the cognitive inclusion criteria is memory-related, in theory our study population is at risk of both AD type and vascular cognitive impairment. The mean cognitive performance of our study population was less than 0.5 SD below the average level of a cognitively normal Finnish population. The neuropsychological test battery that was used in the FINGER study includes a set of widely used standard tests, and it is a modified version of test battery that was previously shown to be sensitive to cognitive changes in persons with mild AD [23]. We added standard tests measuring attention and executive functions to the original test battery, in order to better capture also changes typical in vascular cognitive impairment.

In order to have high external validity, *i.e*., that our results would be as generalizable as possible, we kept the exclusion criteria to the minimum and used six study areas that provided good geographical coverage within Finland and comprised both urban and rural areas. The participants were invited from earlier population-based surveys that had very good participation rates and therefore can be considered well representative of the Finnish population. Although we have important information of the characteristics of the screening visit non-attendees, we do not know why they chose not to attend. Both the nature of the trial and characteristics (such as age or health status) of the target population may have played a role. It remains a task for the future to identify if there is something we could do to facilitate participation in future trials and/or when implementing preventive strategies into practice. The differences between the screening visit attendees and non-attendees will not compromise the expected results of our trial, but they are important to identify in order to understand to what extent our results may be generalizable to the population at-large. We hypothesize that the persons with more risk factors would benefit more from the intervention, so given the fact that the study participants had somewhat less risk factors present may underestimate the effect of the intervention in the whole target population.

The FINGER study is using an existing infrastructure and a research framework built for cardiovascular risk factor monitoring to recruit a population that would benefit most from the intervention. Detailed information on earlier lifestyle and vascular factors is available, and differences in these variables can be taken into account in the analyses, which is usually not possible in randomized controlled trials. An extended follow-up of the cohort after the end of the 2 year intervention will provide much needed information on the long-term effects of the 2-year intervention, especially on the incidence of dementia/AD. We will utilize the exceptional opportunities available in Finland, including structured and detailed health registers of high quality, to ensure a complete long-term follow-up on a wide range of outcomes also for the persons who did not participate in the screening or the actual trial, were not included or dropped out during the study.

## 4. Conclusions

Vascular risk factors were more common among those who did not attend the FINGER study screening visit than among those who attended. Nevertheless, as expected from the inclusion criteria the participants had several modifiable risk factors for dementia and cognitive impairment at baseline indicating a window of opportunity for the intervention. The random allocation of participants to the intervention and control groups was successful. The FINGER study is expected to provide important novel information on the effect of lifestyle intervention to prevent cognitive impairment among at risk persons selected from the general population.

## References

[1] Prince M., Bryce R., Albanese E., Wimo A., Ribeiro W., Ferri C.P. (2013). The global prevalence of dementia: A systematic review and metaanalysis. Alzheimers Dement..

[2] Mangialasche F., Kivipelto M., Solomon A., Fratiglioni L. (2012). Dementia prevention: Current epidemiological evidence and future perspective. Alzheimers Res. Ther..

[3] Williams J., Plassman B., Burke J., Holsinger T., Benjamin S. (2010). Preventing Alzheimer’s Disease and Cognitive Decline.

[4] Peters R., Beckett N., Forette F., Tuomilehto J., Clarke R., Ritchie C., Waldman A., Walton I., Poulter R., Ma S. (2008). Incident dementia and blood pressure lowering in the Hypertension in the Very Elderly Trial cognitive function assessment (HYVET-COG): A double-blind, placebo controlled trial. Lancet Neurol..

[5] De Jager C.A., Oulhaj A., Jacoby R., Refsum H., Smith A.D. (2012). Cognitive and clinical outcomes of homocysteine-lowering B-vitamin treatment in mild cognitive impairment: A randomized controlled trial. Int. J. Geriatr. Psychiatry.

[6] Lautenschlager N.T., Cox K.L., Flicker L., Foster J.K., van Bockxmeer F.M., Xiao J., Greenop K.R., Almeida O.P. (2008). Effect of physical activity on cognitive function in older adults at risk for Alzheimer disease: A randomized trial. JAMA.

[7] Willis S.L., Tennstedt S.L., Marsiske M., Ball K., Elias J., Koepke K.M., Morris J.N., Rebok G.W., Unverzagt F.W., Stoddard A.M. (2006). Long-term effects of cognitive training on everyday functional outcomes in older adults. JAMA.

[8] Tuomilehto J., Lindstrom J., Eriksson J.G., Valle T.T., Hamalainen H., Ilanne-Parikka P., Keinanen-Kiukaanniemi S., Laakso M., Louheranta A., Rastas M. (2001). Prevention of type 2 diabetes mellitus by changes in lifestyle among subjects with impaired glucose tolerance. N. Engl. J. Med..

[9] Prindle J.J., McArdle J.J. (2014). How representative is the ACTIVE sample? A statistical comparison of the ACTIVE sample and the HRS sample. J. Aging Health.

[10] Launer L.J., Wind A.W., Deeg D.J. (1994). Nonresponse pattern and bias in a community-based cross-sectional study of cognitive functioning among the elderly. Am. J. Epidemiol..

[11] Rusanen M., Ngandu T., Laatikainen T., Tuomilehto J., Soininen H., Kivipelto M. (2013). Chronic obstructive pulmonary disease and asthma and the risk of mild cognitive impairment and dementia: A population based CAIDE study. Curr. Alzheimer Res..

[12] Harris T.J., Victor C.R., Carey I.M., Adams R., Cook D.G. (2008). Less healthy, but more active: Opposing selection biases when recruiting older people to a physical activity study through primary care. BMC Public Health.

[13] Van Heuvelen M.J., Hochstenbach J.B., Brouwer W.H., de Greef M.H., Zijlstra G.A., van Jaarsveld E., Kempen G.I., van Sonderen E., Ormel J., Mulder T. (2005). Differences between participants and non-participants in an RCT on physical activity and psychological interventions for older persons. Aging Clin. Exp. Res..

[14] Ives D.G., Traven N.D., Kuller L.H., Schulz R. (1994). Selection bias and nonresponse to health promotion in older adults. Epidemiology.

[15] Kivipelto M., Solomon A., Ahtiluoto S., Ngandu T., Lehtisalo J., Antikainen R., Backman L., Hanninen T., Jula A., Laatikainen T. (2013). The finnish geriatric intervention study to prevent cognitive impairment and disability (FINGER): Study design and progress. Alzheimers Dement..

[16] Vartiainen E., Laatikainen T., Peltonen M., Juolevi A., Mannisto S., Sundvall J., Jousilahti P., Salomaa V., Valsta L., Puska P. (2010). Thirty-five-year trends in cardiovascular risk factors in Finland. Int. J. Epidemiol..

[17] Saaristo T., Peltonen M., Keinanen-Kiukaanniemi S., Vanhala M., Saltevo J., Niskanen L., Oksa H., Korpi-Hyovalti E., Tuomilehto J. (2007). National type 2 diabetes prevention programme in Finland: FIN-D2D. Int. J. Circumpolar Health.

[18] Peltonen M., Harald K., Männistö S., Saaikoski S., Peltomäki P., Lund L., Sundvall J., Juolevi A., Laatikainen T., Aldén-Nieminen H. (2008). Kansallinen FINRISKI 2007—Terveystutkimus—Tutkimuksen Toteutus ja Tulokset.

[19] Kivipelto M., Ngandu T., Laatikainen T., Winblad B., Soininen H., Tuomilehto J. (2006). Risk score for the prediction of dementia risk in 20 years among middle aged people: A longitudinal, population-based study. Lancet Neurol..

[20] Morris J.C., Heyman A., Mohs R.C., Hughes J.P., van Belle G., Fillenbaum G., Mellits E.D., Clark C. (1989). The Consortium to Establish a Registry for Alzheimer’s Disease (CERAD). Part I. Clinical and neuropsychological assessment of Alzheimer’s disease. Neurology.

[21] Pulliainen V., Hänninen T., Hokkanen L., Tervo S., Vanhanen M., Pirttilä T., Soininen H. (2007). Norms for use of the CERAD test battery in Finland. Suom. Lääkäril. (Finn. Med. J.).

[22] Guralnik J.M., Simonsick E.M., Ferrucci L., Glynn R.J., Berkman L.F., Blazer D.G., Scherr P.A., Wallace R.B. (1994). A short physical performance battery assessing lower extremity function: Association with self-reported disability and prediction of mortality and nursing home admission. J. Gerontol..

[23] Harrison J., Minassian S.L., Jenkins L., Black R.S., Koller M., Grundman M. (2007). A neuropsychological test battery for use in Alzheimer disease clinical trials. Arch. Neurol..

[24] Wechsler D. (1998). WMS-III—Administration and Scoring Manual.

[25] Van der Elst W., Van Boxtel M.P., Van Breukelen G.J., Jolles J. (2006). The concept shifting test: Adult normative data. Psychol. Assess..

[26] Reitan R.M. (1958). Validity of the trail making test as an indicator of organic brain damage. Percept. Mot. Skills.

[27] Golden C. (1978). Stroop Color and Word Test: A Manual for Clinical and Experimental Uses.

[28] Van der Elst W., van Boxtel M.P., van Breukelen G.J., Jolles J. (2006). The letter digit substitution test: Normative data for 1858 healthy participants aged 24–81 from the Maastricht Aging Study (MAAS): Influence of age, education, and sex. J. Clin. Exp. Neuropsychol..

[29] Borodulin K., Levälahti E., Saarikoski L., Lund L., Juolevi A., Grönholm M., Jula A., Laatikainen T., Männistö S., Peltonen M. (2013). Kansallinen FINRISKI 2012—Terveystutkimus—Osa 2: Tutkimuksen taulukkoliite. Terveyden ja Hyvinvoinnin Laitos.

